# Feature Reviews in Pharmaceutical Technology

**DOI:** 10.3390/ph16101336

**Published:** 2023-09-22

**Authors:** Silviya Petrova Zustiak, Era Jain

**Affiliations:** 1 Department of Biomedical Engineering, Saint Louis University, St. Louis, MO 60103, USA; 2 Department of Biomedical and Chemical Engineering, Syracuse University, Syracuse, NY 13244, USA

We are excited to present the Special Issue, “Feature Reviews in Pharmaceutical Technology”, aiming to highlight exciting developments in pharmaceutical technologies. With the continual discovery of a plethora of new drug candidates, both small-molecule drugs and large biomolecules, such technologies are becoming essential for the advancement of patient treatments. This issue features eleven review papers broadly classified into formulation development and drug delivery devices. These technologies address important issues of improving drug bioavailability, decreasing treatment toxicity and side effects, and enhancing treatment efficacy to both cure patients and improve their quality of life.

Several reviews focus on formulation and drug delivery devices with a focus on the delivery device. A review by Stealey et al. [1] describes the use of nanosilicates and nanosilicate–hydrogel composites for the sustained and localized delivery of small-molecule drugs as well as large biomolecules. The charged nanosilicate faces allow for drug adsorption via electrostatic interactions and subsequent sustained release, while preserving therapeutic structure and bioactivity [1]. A paper by Oliveira et al. [2] describes a patient-centric design of topical dermatological medicines to improve patient compliance. In a patient-centered approach, the needs and preferences of the patient as well as the needs associated with the disease are all taken into account when designing new topical vehicles meant to improve patient satisfaction and adherence [2]. Yet, another review by Wang et al. [3] describes polymeric micellar formulations used in cancer therapy, which can target specific tissues and prolong therapeutic bioavailability. The review describes polymers used in micelle formulations, tailoring them to be responsive to stimuli for drug delivery applications [3]. Another review by Franc et al. [4] focuses on Enteric Capsule Drug Delivery Technology, which is used to prepare hard enteric capsules meant to survive the acidic stomach environment and slowly erode when passing through the digestive tract. Such capsules could be used for individual therapy or the clinical evaluation of therapeutic substances such as fecal material or probiotics, when administered orally [4]. Lastly, a review from Litvinova et al. [5] offers a patent landscape analysis for pills with digital sensors, showing an increase in patents for applications such as mobile clinical monitoring, smart drug delivery, and endoscopy diagnostics. Such pills have been used to treat various diseases, such as mental health issues, HIV/AIDS, pain, cardiovascular diseases, diabetes, cancer, tuberculosis, etc. [5].

Other feature reviews focus on the co-formulants and process parameters used to design drug delivery devices which influence their performance. For example, a review by Hsieh et al. [6] focuses on oral delivery via self-microemulsifying systems, which improve drug solubility and absorption by enabling drug self-emulsification in a combination of oil, surfactant, and co-surfactant. The authors highlight the use of “design of experiments” systematic approaches for formulation optimization for drugs in different excipients [6]. Another review by Kim et al. [7] focuses on gastrointestinal permeation enhancers for the development of oral peptide products, particularly semaglutide and octreotide, where an oral dosage form of semaglutide is used for the treatment of type 2 diabetes and octreotide capsule is used for the treatment of acromegaly. The article discusses the permeation properties of sodium salcaprozate and medium-chain fatty acids, sodium caprate and sodium caprylate, focusing on transient permeation enhancer technology and gastrointestinal permeation enhancement technology [7].

Some reviews focus on delivery devices for specific diseases and take a disease-centered approach. For example, a paper by Nair et al. [8] focuses on KIF1A-associated neurological diseases, which are caused by changes in the KIF1A microtubule motor protein as a result of gene mutations. While there is no treatment for this class of diseases, the review discusses the promise of experimental gene therapy approaches, such as gene replacement, gene knockdown, symptomatic gene therapy, and cell suicide gene therapy [8]. Another example by Li et al. [9] focuses on diabetic kidney disease, which is a leading cause of end-stage kidney disease in the world. Specifically, the paper discusses proximal tubule cell-targeted drug delivery strategies, such as prodrugs, large molecule carriers and nanoparticles; the proximal tubule is critical for disease progression, and hence, is a worthy target [9].

Several papers focus on formulation development for specific therapeutics, where the focus is on some critical therapeutics. For example, a review by Lee et al. [10] focuses on formulations and drug delivery devices meant to improve the efficacy and reduce the toxicity of the chemotherapeutic doxorubicin. The discussed technologies, such as liposomes, polymeric micelles, polymeric nanoparticles, and polymer–drug conjugates, are currently in clinical use or clinical trials [10]. Another paper by Bonaccorso et al. [11] focuses on the therapeutic potential of formulations for carnosine, which is an endogenous dipeptide with anti-oxidant and ant-inflammatory properties and is found in tissues with a high metabolic rate. Because this dipeptide is rapidly hydrolyzed in plasma, drug modifications and drug delivery systems have been employed to improve its bioavailability as well as to enhance other pharmacological properties, such as blood–brain barrier transport, biological activity, or transport to different tissues [11].

In summary, the papers in this Special Issue highlight the diversity of approaches in formulations and drug delivery device development as well as the diversity of applications that benefit from the advancement of such pharmaceutical technologies. The common goal for such technologies is to improve drug bioavailability, which translates to better efficacy and patient outcomes. In certain instances, as exemplified above, formulations and drug delivery devices can be used to provide targeted treatment, which reduces side effects. They can also be used to provide sustained release, which reduces the treatment frequency and improves patient compliance, ultimately improving the patient’s quality of life. With the discovery of newer biotherapeutics, such as the peptides highlighted above, formulations and drug delivery devices have become essential for countering the usually unfavorable pharmacokinetic drug profiles. Hence, innovation in the realm of formulations and drug delivery devices will expand therapy options for patients and has the potential to cure previously incurable diseases.
